# Association of serum creatinine to cystatin C to waist circumference ratios and hypertension: evidence from China health and retirement longitudinal study

**DOI:** 10.3389/fendo.2024.1375232

**Published:** 2024-05-01

**Authors:** Yang Yang, Qi Sun, Shuang Ma, Xiaodan Li, Xinmiao Lang, Qi Zhang

**Affiliations:** ^1^ Department of Pediatrics, China-Japan Friendship Hospital, Beijing, China; ^2^ Nursing Department, The Fourth Affiliated Hospital of China Medical University, Shengyang, China; ^3^ Graduate School of Peking Union Medical College, Chinese Academy of Medical Sciences, Beijing, China

**Keywords:** serum creatinine, cystatin C, waist circumference, hypertension, abdominal obesity

## Abstract

**Background:**

The objective of this study was to explore the association between the ratio of serum creatinine to cystatin C to waist circumference (CCR/WC) and hypertension.

**Methods:**

The study utilized data extracted from the China Health and Retirement Longitudinal Study. In the cross-sectional analysis, logistic regression analyses were employed to examine the association between the CCR/WC ratio and hypertension. By utilizing restricted cubic splines, potential non-linear associations between the CCR/WC ratio and hypertension were explored. In the longitudinal analysis, the association between CCR/WC quartiles (Q1–Q4) and the risk of new-onset hypertension was evaluated by Cox proportional-hazards models.

**Results:**

In total, 7,253 participants were enrolled. The study unveiled an inverse association with hypertension, demonstrating an odds ratio (OR) of 0.29 (95% confidence interval [CI]: 0.23–0.37, *P* < 0.001). Among males, an OR of 0.38 (95% CI: 0.25–0.58, *P* < 0.001) was observed, while among females, an OR of 0.41 (95% CI: 0.28–0.60, *P* < 0.001) was noted. There was an absence of a nonlinear association between the CCR/WC ratio and hypertension. Cox regression analysis unveiled a reduced risk of hypertension in Q3 (Hazard ratios [HR]: 0.69, 95% CI: 0.58–0.82, *P* < 0.001) and Q4: (HR: 0.70, 95% CI: 0.59–0.83, *P* < 0.001) in compared to the Q1 of the CCR/WC ratio, and sex-specific analysis yielded consistent results.

**Conclusion:**

This study emphasizes the potential association between an elevated CCR/WC ratio and a reduced risk of hypertension.

## Introduction

As rapid socio-economic development unfolds, the aging of the Chinese population is further exacerbated (1). The considerable expansion of the aging population will significantly amplify the societal burden of disease and healthcare requirements (2, 3), with particular emphasis on the pervasive attention given to hypertension among these chronic conditions. Hypertension is a noteworthy global public health concern, with a relatively elevated prevalence of 15.4% in the elderly population (4). Unmanaged hypertension can result in severe health complications, encompassing conditions such as heart disease, stroke, renal disorders, visual impairments, among others (5–7). Early identification and intervention of hypertension are crucial.

The pathogenesis of hypertension remains incompletely elucidated, and the risk of hypertension is linked to a variety of factors, including, age, sex, dietary habits, weight, physical activity, smoking, and drinking (8). Abdominal obesity emerges as a significant risk factor for hypertension (9), and leads to the accumulation of fat around visceral organs, known as visceral fat, which can produce various bioactive substances, influencing metabolism and increasing the risk of hypertension (10, 11). Research findings indicate an association between sarcopenia and hypertension (12). The presence of sarcopenia further contributes to the occurrence of obesity, as sarcopenia leads to reduced physical activity and an increased risk of obesity, a condition referred to as sarcopenic obesity (13). The presence of sarcopenic obesity independently represents a risk factor for hypertension (14). Therefore, in assessing the risk of hypertension, mere estimation of obesity alone falls short; consideration should also be given to sarcopenia.

Creatinine is a waste product generated from muscle metabolism and is primarily excreted from the body through the kidneys (15). Cystatin C is a petite protein synthesized by nucleated cells in the body at a consistently constant rate (16). Previous studies have recognized the creatinine to cystatin C ratio (CCR) not only as an alternative marker for sarcopenia and muscle mass assessment (17–20), but also as being associated with mortality among patients in intensive care (21), as well as with low handgrip strength in older adults (22). Recent research has unveiled a certain correlation between the CCR ratio and hypertension (23). Furthermore, waist circumference (WC) measurement is commonly utilized to evaluate abdominal obesity, where an increased waist circumference may signify a higher accumulation of fat in the abdominal area (24). Compared to the body mass index (BMI), WC serves as a convenient measurement for assessing fat distribution, and exhibits a stronger correlation with visceral adipose tissue (25).

Thus, this study hypothesizes a connection between the CCR to WC ratio (CCR/WC) and hypertension. The principal aim of this study is to examine the association between the CCR/WC and hypertension among elderly Chinese adults, employing both cross-sectional and longitudinal research study design. The results derived from this study will assist physicians in gaining a more comprehensive understanding of whether patients belong to the high-risk category for hypertension, thereby facilitating the implementation of suitable interventions.

## Materials and methods

### Study population

The dataset employed in this study was sourced from the China Health and Retirement Longitudinal Study (CHARLS). CHARLS is a comprehensive longitudinal research initiative, and comprised of 10,257 households and 17,708 middle-aged and elderly participants from 450 villages or urban communities across 150 counties or districts in 28 provinces. It serves as a valuable resource for understanding health and retirement dynamics in China. The detailed information on the sampling design of CHARLS has been published previously (26). CHARLS initiated with a baseline survey in 2011, followed by biennial face-to-face individual interviews, culminating in four waves of follow-up assessments through 2018.

**Figure 1:**
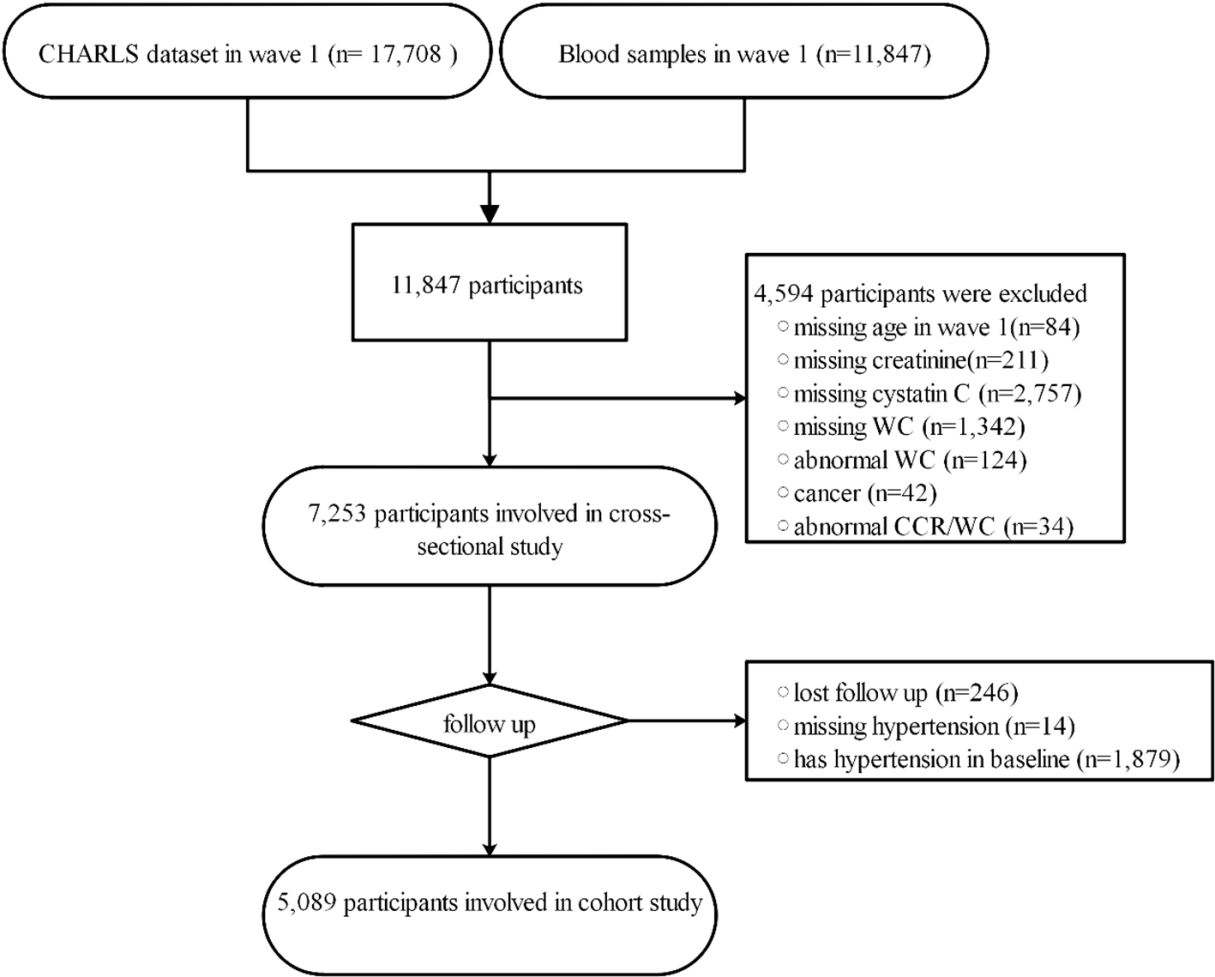
The workflow of study. CCR/WC: Ratio of Serum Creatinine to Cystatin C to Waist Circumference; CHARLS, China Health and Retirement Longitudinal Study; WC, waist circumference.

This study comprises two components. In the cross-sectional part, 4,594 participants were excluded based on specific criteria. These criteria included missing age in wave 1 (n=84), absence of creatinine data (n=211), lack of cystatin C information (n=2,757), missing WC data (n=1,342), abnormal WC (n=124), cancer (n=42), and absence of CCR/WC information (n=34). Ultimately, 7,253 participants were included in the cross-sectional study. Abnormal WC and CCR/WC were defined as values greater than three times the interquartile range. In the second part, a longitudinal cohort analysis, we excluded 2,139 participants based on the following criteria: lost follow-up (n=246), missing hypertension (n=14), and having hypertension at baseline (n=1,879). A total of 5,089 participants were included in the cohort study. The study’s flowchart is depicted in **Figure 1**.

The study design of CHARLS received approval from the Peking University Ethics Review Committee (IRB-00001052-11014). All participants provided informed consent before the initiation of interviews. This study constitutes a secondary analysis based on existing data, and as such, ethical approval and informed consent were not required.

### CCR/WC measurement

Blood samples were collected following an overnight fast. Creatinine measurements were performed utilizing the rate-blanked and compensated Jaffe creatinine assay. Cystatin C measurements were performed using immunoturbidimetric assay with particle enhancement. Comprehensive assay protocols are available in the CHARLS Blood Test Data User Manual (https://charls.charlsdata.com).

The procedure for measuring WC involved placing a measuring tape horizontally at the level of the participant’s waist, aligned with the navel, while the participant stood still, maintained calm breathing, and held their breath at the end of exhalation. The CCR was determined by dividing the creatinine value (mg/dL) by the cystatin C value (mg/L) and multiplying the result by 100, and the CCR/WC was calculated by dividing the CCR by the WC in centimeters (27). The ratio of CCR/WC was obtained at baseline in 2011.

### Hypertension assessment

The assessment of hypertension was conducted through self-reported questionnaire interviews, wherein all participants were asked to respond to the question: ‘Have you been diagnosed with hypertension by a doctor?’

### Covariates

The study encompassed various covariates, including age (in years), sex, residence, marital status, and educational level (elementary school or below/high school or below/vocational training or below/college or below), smoking status (yes/no), drinking (yes/no), activities of daily living (ADL), and the occurrence of diverse chronic conditions, including diabetes (yes/no), lung disease (yes/no), cardiovascular disease (yes/no), stroke (yes/no), kidney disease (yes/no), and asthma (yes/no). ADL was characterized by self-reported challenges and the requirement for assistance in a minimum of one ADL, encompassing tasks like dressing, bathing, eating, mobility (getting in and out of bed), toileting, and bowel control. Moreover, this study incorporated the following covariates related to glucose metabolism (blood glucose and glycated hemoglobin [HbA1c]), lipid profiles (total cholesterol [TC], low-density lipoprotein cholesterol [LDL-C], high-density lipoprotein cholesterol [HDL-C], and triglycerides [TG]), and uric acid levels (UA).

### Statistical analysis

Continuous variables, following a normal distribution, are reported as mean ± standard deviation (SD). Between-group differences in continuous variables were analyzed using either the t-test or Wilcoxon test. Categorical variables are presented as counts (percentages), and group differences for categorical variables were assessed through the chi-square test or Fisher’s exact test.

In the baseline cross-sectional analysis, the link between hypertension and CCR/WC was examined using a logistic regression model, controlling for potential confounding factors, and odds ratios (ORs) were calculated, accompanied by their respective 95% confidence intervals (CIs). The selection of confounding factors was determined based on prior knowledge and meaningful results from univariate analysis (*P* < 0.05). Restricted cubic spline analysis was employed to explore possible non-linear associations between the CCR/WC with hypertension, aiming to identify potential inflection points and elucidate curvature patterns. Regarding the sensitivity analysis, we excluded individuals with kidney conditions, chronic lung conditions, and asthma at baseline to facilitate a reassessment of the association between hypertension and CCR/WC.

In the cohort study, participants with a pre-existing diagnosis of hypertension were excluded from the analysis. Data on the development of hypertension during follow-up were documented at follow-up visits 2 to visits 4. According to quartiles of CCR/WC, the CCR/WC was divided into four groups. The Kaplan-Meier method was employed to analyze the survival probabilities across different quartiles of the CCR/WC. Additionally, Cox proportional hazards models, controlling for confounding factors, were utilized to assess the association between CCR/WC quartiles with the risk of developing hypertension. Hazard ratios (HRs) with 95% Cis were calculated, offering a quantitative representation of the association between CCR/WC quartiles and hypertension risk.

Statistical analyses were performed using the R software (version 4.1.3). Significance testing was conducted with two-tailed *P* values, where a threshold of <0.05 denoted statistical significance.

## Results

### Participant characteristics

In the present study, 7,253 individuals participated in the cross-sectional analysis, as outlined in **Table 1**. The mean age was 59.98 years with a standard deviation of 10.19 years. Overall, 46.2% were male, and 53.8% were female. The proportion of participants residing in urban communities was 34.7%, while those in rural villages accounted for 65.3%. Hypertensive individuals comprised 27.3% of the total population. The proportion of participants with education levels beyond high school and vocational training was lower in the lowest quartile (Q1) (5.2%) and higher in the highest quartile (Q4) (9.9%). Most participants had education levels below elementary school. The smoking prevalence was lower in the lowest quartile (18.3%) and higher in the highest quartile (39.4%). Drinking was less common in the lowest quartile (25.6%) and more prevalent in the highest quartile (47.7%). The proportion of hypertensive individuals was lower in the CCR/WC lowest quartile (20.3%) and higher in the Q4 quartile (79.7%).

**Table 1:** Baseline characteristics of participants.

	level	all (n=7253)	CCR/WC				*P*
			Q1 (n=1822)	Q2 (n=1822)	Q3 (n=1821)	Q4 (n=1788)	
Age (years)		59.98 (10.19)	63.29 (10.65)	60.38 (10.06)	59.02 (9.63)	57.17 (9.39)	< 0.001
Sex	Male	3352 (46.2)	449 (24.6)	771 (42.3)	988 (54.3)	1144 (64.0)	< 0.001
	Female	3901 (53.8)	1373 (75.4)	1051 (57.7)	833 (45.7)	644 (36.0)	
Residence	Urban Community	2514 (34.7)	655 (35.9)	597 (32.8)	617 (33.9)	645 (36.1)	0.098
	Rural Village	4739 (65.3)	1167 (64.1)	1225 (67.2)	1204 (66.1)	1143 (63.9)	
Married	No	1312 (18.1)	429 (23.5)	327 (17.9)	299 (16.4)	257 (14.4)	< 0.001
	Yes	5941 (81.9)	1393 (76.5)	1495 (82.1)	1522 (83.6)	1531 (85.6)	
Educational	Less than lower secondary	6616 (91.2)	1716 (94.2)	1664 (91.3)	1648 (90.5)	1588 (88.8)	< 0.001
	Upper secondary & vocational training	553 (7.6)	94 (5.2)	141 (7.7)	141 (7.7)	177 (9.9)	
	Tertiary	84 (1.2)	12 (0.7)	17 (0.9)	32 (1.8)	23 (1.3)	
Smoking	No	5044 (69.8)	1482 (81.7)	1303 (71.8)	1179 (65.1)	1080 (60.6)	< 0.001
	Yes	2179 (30.2)	332 (18.3)	513 (28.2)	631 (34.9)	703 (39.4)	
Drinking	No	4466 (61.6)	1354 (74.4)	1141 (62.7)	1035 (56.9)	936 (52.3)	< 0.001
	Yes	2783 (38.4)	467 (25.6)	679 (37.3)	785 (43.1)	852 (47.7)	
WC (cm)		85.21 (10.22)	91.16 (10.22)	86.20 (9.04)	83.12 (9.07)	80.26 (9.20)	< 0.001
PG (mg/dL)		110.23 (36.69)	110.99 (34.79)	108.16 (29.92)	110.12 (37.50)	111.69 (43.35)	0.024
TC (mg/dL)		193.52 (38.63)	192.81 (40.21)	191.79 (38.00)	192.12 (37.00)	197.42 (39.03)	< 0.001
TG (mg/dL)		131.02 (99.37)	132.83 (113.14)	127.54 (85.07)	123.42 (83.29)	140.46 (111.47)	< 0.001
LDL-C (mg/dL)		116.76 (35.19)	117.81 (36.19)	116.70 (34.94)	116.86 (33.95)	115.67 (35.66)	0.338
HDL-C (mg/dL)		51.27 (15.27)	49.85 (14.62)	51.14 (14.65)	51.97 (15.50)	52.12 (16.17)	< 0.001
HbA1c (mg/dL)		5.27 (0.82)	5.33 (0.83)	5.26 (0.78)	5.24 (0.82)	5.23 (0.83)	0.001
UA (mg/dL)		4.46 (1.27)	4.26 (1.18)	4.39 (1.27)	4.50 (1.26)	4.70 (1.33)	< 0.001
Creatinine (mg/dL)		0.79 (0.25)	0.68 (0.17)	0.76 (0.18)	0.81 (0.18)	0.90 (0.37)	< 0.001
Cystatin C (mg/L)		1.03 (0.29)	1.14 (0.31)	1.05 (0.24)	1.00 (0.22)	0.91 (0.32)	< 0.001
CCR/WC		0.93 (0.23)	0.66 (0.09)	0.84 (0.04)	0.99 (0.05)	1.24 (0.16)	< 0.001
ADL	No	5926 (82.5)	1358 (75.1)	1492 (82.8)	1534 (85.2)	1542 (87.0)	< 0.001
	Yes	1259 (17.5)	451 (24.9)	311 (17.2)	266 (14.8)	231 (13.0)	
Hypertension	No	5250 (72.7)	1171 (64.6)	1309 (72.1)	1352 (74.6)	1418 (79.7)	< 0.001
	Yes	1970 (27.3)	642 (35.4)	507 (27.9)	460 (25.4)	361 (20.3)	
Diabetes	No	6751 (93.6)	1658 (91.7)	1705 (94.2)	1704 (94.1)	1684 (94.5)	0.001
	Yes	461 (6.4)	151 (8.3)	105 (5.8)	107 (5.9)	98 (5.5)	
Lung disease	No	6463 (89.3)	1612 (88.7)	1618 (88.9)	1633 (89.9)	1600 (89.8)	0.584
	Yes	772 (10.7)	205 (11.3)	201 (11.1)	184 (10.1)	182 (10.2)	
CVD	No	6324 (87.6)	1524 (84.1)	1591 (87.7)	1593 (87.9)	1616 (90.6)	< 0.001
	Yes	899 (12.4)	289 (15.9)	223 (12.3)	219 (12.1)	168 (9.4)	
Stroke	No	7039 (97.3)	1751 (96.3)	1759 (96.9)	1784 (98.1)	1745 (97.8)	0.003
	Yes	197 (2.7)	67 (3.7)	56 (3.1)	34 (1.9)	40 (2.2)	
Kidney	No	6784 (94.1)	1714 (94.5)	1714 (94.7)	1698 (93.8)	1658 (93.3)	0.259
	Yes	428 (5.9)	100 (5.5)	96 (5.3)	113 (6.2)	119 (6.7)	
Asthma	No	6863 (95.0)	1711 (94.2)	1723 (94.9)	1726 (95.1)	1703 (95.6)	0.253
	Yes	365 (5.0)	106 (5.8)	92 (5.1)	89 (4.9)	78 (4.4)	

HDL-C, High-density lipoprotein cholesterol; LDL-C, Low-density lipoprotein cholesterol; TC, Total cholesterol; TG, Triglycerides; UA, Uric acid; HbA1c, Glycated hemoglobin A1c; WC, Waist circumference; PG, Plasma glucose; ADL, Activities of daily living; CVD, Cardiovascular disease; The CCR/WC ratio is determined by dividing the ratio of Creatinine to Cystatin C by the waist circumference, and then multiplying the result by 100.

### Association of CCR/WC with hypertension at baseline

**Table 2** illustrates the association between CCR/WC and baseline hypertension. In the crude model, CCR/WC showed a significant inverse association with hypertension (OR: 0.29, 95% CI: 0.23–0.37, *P* < 0.001). After adjusting for various covariates in model 1, the association remained significant (OR: 0.46, 95% CI: 0.35–0.59, *P* < 0.001). Further adjustment in model 2 yielded an OR of 0.40 (95% CI: 0.31–0.53, *P* < 0.001). Among males, the crude model demonstrated a significant inverse association (OR: 0.30, 95% CI: 0.21–0.44, *P* < 0.001). Adjusting for covariates in model 1 resulted in an OR of 0.45 (95% CI: 0.30–0.67, *P* < 0.001). Model 2, with further adjustments, showed an OR of 0.38 (95% CI: 0.25–0.58, *P* < 0.001). Among females, the crude model yielded an OR of 0.28 (95% CI: 0.20–0.39, *P* < 0.001). In model 1, adjusting for covariates, the OR was 0.45 (95% CI: 0.31–0.64, *P* < 0.001). Model 2, with additional adjustments, showed an OR of 0.41 (95% CI: 0.28–0.60, *P* < 0.001). Sensitivity analysis revealed consistent findings with the exclusion of participants having baseline kidney disease, chronic lung disease, or asthma in **Supplementary Table 1**.

**Table 2:** Association between CCR/WC ratio and hypertension at baseline.

		OR (95%CI)	*P*
All			
	Crude model	0.29 (0.23, 0.37)	< 0.001
	Adjusted model 1	0.46 (0.35, 0.59)	< 0.001
	Adjusted model 2	0.40 (0.31, 0.53)	< 0.001
Male			
	Crude model	0.30 (0.21, 0.44)	< 0.001
	Adjusted model 1	0.45 (0.30, 0.67)	< 0.001
	Adjusted model 2	0.38 (0.25, 0.58)	< 0.001
Female			
	Crude model	0.28 (0.20, 0.39)	< 0.001
	Adjusted model 1	0.45 (0.31, 0.64)	< 0.001
	Adjusted model 2	0.41 (0.28, 0.60)	< 0.001

OR, Odds Ratio; CI, Confidence Interval.

Crude model: Logistic regression. Model 1: Crude model adjusted for baseline demographics (age, sex, residential status, marital status, education level), lifestyle factors (smoking, drinking), and health status (activities of daily living, diabetes, lung disease, cardiovascular disease, stroke, kidney disease, asthma). Model 2: Model 1 additionally adjusted for key biomarkers, including fasting plasma glucose, lipid profile (total cholesterol, triglycerides, low-density lipoprotein cholesterol, high-density lipoprotein cholesterol), glycated hemoglobin, and uric acid.

**Figure 2:**
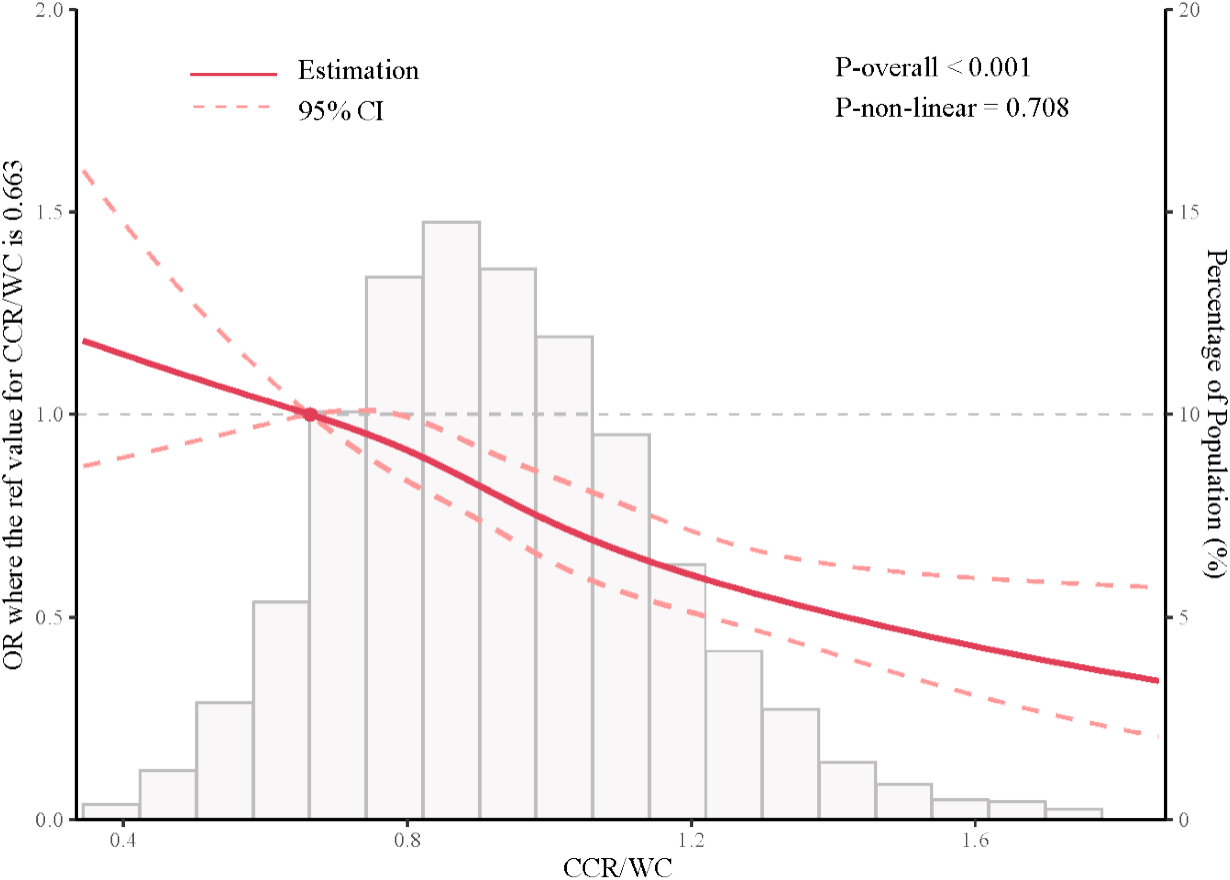
Restricted cubic spline analysis depicting the association between CCR/WC ratio and hypertension. CCR/WC: serum creatinine to cystatin C to waist circumference ratio.

The findings of RCS indicated that there was no non-linear association between the hypertension and CCR/WC. As the CCR/WC increased, a decrease in the OR was observed, indicating a protective effect of the CCR/WC ratio against hypertension, particularly when the CCR/WC ratio exceeded 0.663, as illustrated in **Figure 2**.

### Risk of new onset hypertension of CCR/WC quartiles

**Figure 3:**
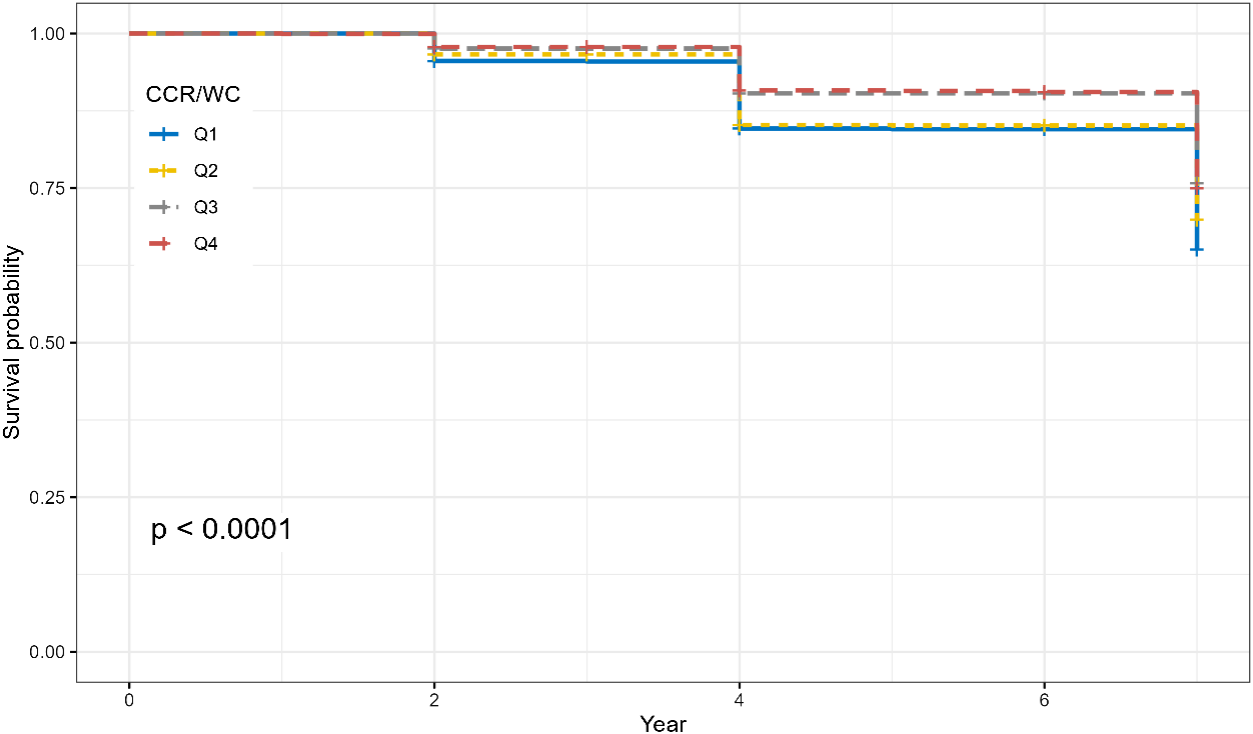
Survival curves illustrating the impact of CCR/WC quartiles on the risk of hypertension. The *P* value was calculated by the log-rank test.

A longitudinal analysis was conducted with 5,089 participants free from hypertension at baseline, exploring the association between the CCR/WC and the onset of new hypertension. The CCR/WC was stratified into four groups according to quartiles, and survival analysis demonstrated notable variations in survival rates among the distinct CCR/WC groups (**Figure 3**, *P* < 0.001).

Furthermore, the results from the Cox regression model demonstrated that in the overall population, individuals in the second quartile (Q2) had a hazard ratio (HR) of 0.84 (95% CI: 0.73–0.98, *P* = 0.026). Participants in the third quartile (Q3) demonstrated a significantly lower risk with an HR of 0.64 (95% CI: 0.55, 0.75; *P* < 0.001). The fourth quartile (Q4) also displayed a reduced risk with an HR of 0.66 (95% CI: 0.56–0.77, *P* < 0.001). After adjusting for multiple covariates, including age, sex, residence, marital status, education, lifestyle factors, and comorbidities, the inverse association persisted. Participants in Q3 still exhibited a reduced risk of new-onset hypertension with an HR of 0.68 (95% CI: 0.57–0.80, *P* < 0.001). Further adjustments for metabolic and biochemical factors strengthened the observed associations. In the overall population, Q3 and Q4 maintained a reduced risk of new-onset hypertension, with HRs of 0.69 (95% CI: 0.58–0.82, *P* < 0.001) and 0.70 (95% CI: 0.59–0.83, *P* < 0.001), respectively. Sex-specific analysis yielded consistent results, indicating that the inverse association between CCR/WC and new-onset hypertension remained robust across different models (**Table 3**).

**Table 3:** Association between baseline CCR/WC and new-onset hypertension in longitudinal cohort analysis.

CCR/WC	All				Male	
	HR (95%CI)	*P*	HR (95%CI)	*P*	HR (95%CI)	*P*
Crude model						
Q1	Ref		Ref		Ref	
Q2	0.84 (0.73, 0.98)	0.026	0.97 (0.74, 1.27)	0.820	0.78 (0.65, 0.93)	0.007
Q3	0.64 (0.55, 0.75)	< 0.001	0.63 (0.48, 0.82)	0.001	0.66 (0.54, 0.81)	< 0.001
Q4	0.66 (0.56, 0.77)	< 0.001	0.74 (0.57, 0.96)	0.021	0.54 (0.43, 0.69)	< 0.001
Adjusted model 1						
Q1	Ref		Ref		Ref	
Q2	0.88 (0.76, 1.03)	0.113	0.95 (0.72, 1.25)	0.700	0.87 (0.72, 1.06)	0.165
Q3	0.68 (0.57, 0.80)	< 0.001	0.64 (0.48, 0.84)	0.002	0.75 (0.61, 0.94)	0.010
Q4	0.74 (0.62, 0.87)	< 0.001	0.80 (0.62, 1.05)	0.109	0.65 (0.51, 0.83)	0.001
Adjusted model 2						
Q1	Ref		Ref		Ref	
Q2	0.90 (0.77, 1.05)	0.167	0.95 (0.72, 1.26)	0.735	0.88 (0.73, 1.07)	0.198
Q3	0.69 (0.58, 0.82)	< 0.001	0.65 (0.49, 0.86)	0.002	0.77 (0.62, 0.95)	0.017
Q4	0.70 (0.59, 0.83)	< 0.001	0.77 (0.59, 1.01)	0.063	0.62 (0.48, 0.79)	< 0.001

HR, Hazard Ratios; CI, Confidence Intervals; The CCR/WC ratio was divided into four quartiles (Q1 to Q4).

Crude Model: Cox regression model. Adjusted Model 1: Crude model adjusted for baseline demographics, lifestyle factors (smoking, drinking), and health status (body mass index, activities of daily living, diabetes, lung disease, cardiovascular disease, stroke, kidney disease, and asthma). Adjusted Model 2: Model 1 further adjusted for key biomarkers, including fasting plasma glucose, lipid profile (total cholesterol, triglycerides, low-density lipoprotein cholesterol, high-density lipoprotein cholesterol), glycated hemoglobin, and uric acid.

## Discussion

This study, based on a population of elderly Chinese individuals, utilized both cross-sectional and longitudinal cohort designs to explore the association between hypertension and CCR/WC. The study results suggest that the CCR/WC serves as a protective factor against hypertension, which was further corroborated through longitudinal analysis. Sex-specific analysis revealed no significant differences in this protective effect between males and females. Additionally, restricted cubic spline analysis indicated the absence of a non-linear association between hypertension and the CCR/WC.

To our knowledge, this study represents the initial exploration into the association between the risk of hypertension and the CCR/WC. CCR is acknowledged as an indicator of muscle wasting, serving as an alternative measure for assessing muscle mass (28, 29). Prior investigations have proposed cystatin C as a predictive biomarker in pulmonary hypertension (30). However, these investigations did not incorporate the independent influences of muscle mass and adipose tissue measurements when evaluating the risk of hypertension. Prior studies have also suggested a link between abdominal obesity and an elevated risk of hypertension (31). Moreover, obesity may contribute to lipid abnormalities, potentially heightening the risk of conditions related to hypertension (32). Hence, we utilized the CCR/WC to discern between lean body mass and abdominal obesity in this investigation. Our study reveals that a higher CCR/WC ratio is associated with a decreased risk of hypertension development. A cross-sectional study may introduce potential reverse causality. Therefore, we followed up with individuals who did not have hypertension at baseline. The results from longitudinal analysis substantiated a causal link between the CCR/WC and the development of hypertension. The CCR/WC ratio shows potential as a predictive marker for conditions influenced by both muscle mass and obesity. Its validation in various contexts is a prospect for future exploration.

Several potential mechanisms explain the association between CCR/WC, and hypertension. Firstly, Creatinine and cystatin C independently serve as markers of renal health (33). CCR, leveraging both markers, yields a more stable and reliable glomerular filtration rate (GFR) metric, enhancing the accuracy of renal function assessment (34). An elevated level of CCR may suggest an increase in the GFR (35–37), which plays a pivotal role in maintaining fluid balance and regulating blood pressure (38). Enhanced GFR facilitates the effective excretion of sodium and waste, thereby aiding in the prevention of volume overload—a key contributor to the development of hypertension (39). Consequently, the heightened GFR, as indicated by an increased CCR, ostensibly lowers the risk of hypertension through effective fluid balance management and blood pressure regulation. This aspect potentially elucidates a portion of the mechanism underlying the association between the CCR/WC ratio and hypertension. Additionally, muscle tissue is a critical component of the body’s metabolism (40), and CCR/WC indirectly reflects muscle mass. A relatively higher CCR/WC ratio may indicate more muscle mass, which is essential for glucose metabolism and insulin sensitivity (41). Thus, individuals with an elevated CCR/WC may be better equipped to maintain normal metabolism, reducing the risk of hypertension. Finally, abdominal obesity constitutes a risk factor for hypertension (42), and considering it in conjunction with CCR/WC may offer a more thorough comprehension of the pathophysiology underlying hypertension. A higher CCR/WC ratio may reflect lower abdominal fat accumulation, helping to reduce visceral fat accumulation. Visceral fat represents a substantial source of bioactive substances and might contribute to the development of hypertension (43). However, additional research is needed to fully uncover the precise pathways and underlying factors contributing to these associations.

While our study contributes valuable insights, it is important to recognize its limitations. Firstly, although we controlled for numerous covariates, there are still some variables related to hypertension that were not taken into consideration, such as dietary habits (44), albuminuria (45), physical activity (46), and information on antihypertensive treatments such as ACE inhibitors, ARBs, and aldosterone antagonists. In future studies, it will be essential to incorporate these variables into hypertension-related analyses. Secondly, our study is observational, relying on self-reported questionnaires for data collection, including hypertension, which may introduce sampling errors and information bias. Future research should aim for more accurate data collection methods by employing randomized controlled trials, exploring biological mechanisms, and conducting intervention trials to more precisely establish causality. Thirdly, our study includes a higher proportion of residents from rural areas. These individuals may face limitations in accessing healthcare resources and possibly have lower health awareness. To address this, we adjusted for place of residence in our analysis and employed professionally trained personnel to conduct one-on-one interviews. This approach was designed to minimize information bias stemming from definitions of hypertension and awareness of comorbid conditions. Lastly, we utilized four wave data of CHARLS. Subsequent study should involve greater sample sizes, longer term, and multicenter studies to determine the longitudinal changes and development of hypertension associated with CCR/WC ratio. Despite these limitations, our study provides valuable insights into the association between CCR/WC ratio and hypertension in elderly individuals and lays the foundation for further research in this field. Despite these constraints, our study offers significant findings regarding the association between the hypertension and CCR/WC in Chinese older adults, establishing the foundation for further research in this field.

The findings of our study carry noteworthy implications for both clinical practice and public health policy. Firstly, our findings offer clinicians a simple yet potentially predictive tool. The CCR/WC ratio can be utilized for screening and assessing hypertension risk, particularly in the elderly population, who may be at a higher risk for hypertension (47). Secondly, based on the CCR/WC ratio, clinicians can personalize treatment plans more effectively. For hypertensive individuals with higher CCR/WC ratios, a greater emphasis on managing muscle mass and abdominal obesity, possibly through tailored dietary and exercise programs, may be warranted to control hypertension (48). Lastly, the findings of this study bear importance in shaping public health policies. Governments and health authorities may consider incorporating the CCR/WC ratio as part of hypertension risk assessment, including it in routine health check-ups and community health promotion activities. By enhancing public awareness and understanding of the CCR/WC ratio, it is possible to facilitate the early detection and management of hypertension on a broader scale.

## Conclusions

In conclusion, our study illuminates the association between hypertension and CCR/WC in elderly Chinese adults, and an elevated CCR/WC is linked to a diminished risk of hypertension. Our study implies that supervising and enhancing the CCR/WC could be a valuable strategy in the control and management of hypertension.

## Data availability statement

Publicly available datasets were analyzed in this study. This data can be found here: https://charls.charlsdata.com/index/zh-cn.html.

## Ethics statement

The studies involving humans were approved by Peking University Ethics Review Committee (IRB-00001052-11014). The studies were conducted in accordance with the local legislation and institutional requirements. Written informed consent for participation was not required from the participants or the participants’ legal guardians/next of kin in accordance with the national legislation and institutional requirements.

## Author contributions

YY: Methodology, Conceptualization, Writing – original draft, Writing – review & editing. QS: Conceptualization, Writing – original draft, Writing – review & editing. SM: Data curation, Writing – original draft, Writing – review & editing. XDL: Data curation, Writing – original draft, Writing – review & editing. XML: Data curation, Writing – original draft, Writing – review & editing. QZ: Conceptualization, Writing – original draft, Writing – review & editing.
