# Supplementary material for: Association of serum creatinine to cystatin C to waist circumference ratios and hypertension: evidence from China health and retirement longitudinal study
Source: Front Endocrinol (Lausanne). 2024 May 1;15:1375232. doi: 10.3389/fendo.2024.1375232 (PMC11094208; doi:10.3389/fendo.2024.1375232)
Supplement: Supplementary file 1 [file Table_1.docx]

**Supplemental Table 1. Association of CCR/WC with hypertension in sensitivity analysis.**

| Model | OR (95%CI) | *P* |
| --- | --- | --- |
| Individuals with baseline kidney disease, chronic lung conditions, and asthma was excluded. | 0.40 (0.29, 0.54) | < 0.001 |

OR: Odds Ratio; CI: Confidence Interval.
